# Cellular Immune Activation in Cerebrospinal Fluid From Ugandans With Cryptococcal Meningitis and Immune Reconstitution Inflammatory Syndrome

**DOI:** 10.1093/infdis/jiu664

**Published:** 2014-12-09

**Authors:** David B. Meya, Samuel Okurut, Godfrey Zziwa, Melissa A. Rolfes, Melander Kelsey, Steve Cose, Moses Joloba, Prossy Naluyima, Brent E. Palmer, Andrew Kambugu, Harriet Mayanja-Kizza, Paul R. Bohjanen, Michael A. Eller, Sharon M. Wahl, David R. Boulware, Yuka C. Manabe, Edward N. Janoff

**Affiliations:** 1 Infectious Disease Institute; 2 School of Medicine, College of Health Sciences; 3 School of Biomedical Sciences, Microbiology Department, Makerere University; 4 Makerere University Walter Reed Project, KampalaUganda; 5 Medical Research Council/Uganda Virus Research InstituteUganda Research Unit on AIDS, Entebbe; 6 Department of Medicine, Center for Infectious Diseases and Microbiology Translational Research, University of Minnesota, Minneapolis; 7 Mucosal and Vaccine Research Program Colorado, University of Colorado Denver, Aurora; 8 Denver Veterans Affairs Medical Center; 9 Division of Infectious Diseases, Department of Medicine, Johns Hopkins University, Baltimore; 10 US Military HIV Research Program, Walter Reed Army Institute of Research, Silver Spring; 11 Henry M. Jackson Foundation for the Advancement of Military Medicine; 12 National Institute of Dental and Craniofacial Research, National Institutes of Health, Bethesda, Maryland; 13 London School of Hygiene and Tropical Medicine, United Kingdom

**Keywords:** cryptococcal meningitis, cryptococcus, HIV, cerebrospinal fluid, immune responses, cell activation

## Abstract

***Background.*** Human immunodeficiency virus (HIV)-associated cryptococcal meningitis (CM) is characterized by high fungal burden and limited leukocyte trafficking to cerebrospinal fluid (CSF). The immunopathogenesis of CM immune reconstitution inflammatory syndrome (IRIS) after initiation of antiretroviral therapy at the site of infection is poorly understood.

***Methods.*** We characterized the lineage and activation status of mononuclear cells in blood and CSF of HIV-infected patients with noncryptococcal meningitis (NCM) (n = 10), those with CM at day 0 (n = 40) or day 14 (n = 21) of antifungal therapy, and those with CM-IRIS (n = 10).

***Results.*** At diagnosis, highly activated CD8^+^ T cells predominated in CSF in both CM and NCM. CM-IRIS was associated with an increasing frequency of CSF CD4^+^ T cells (increased from 2.2% to 23%; *P* = .06), a shift in monocyte phenotype from classic to an intermediate/proinflammatory, and increased programmed death ligand 1 expression on natural killer cells (increased from 11.9% to 61.6%, *P* = .03). CSF cellular responses were distinct from responses in peripheral blood.

***Conclusions.*** After CM, T cells in CSF tend to evolve with the development of IRIS, with increasing proportions of activated CD4^+^ T cells, migration of intermediate monocytes to the CSF, and declining fungal burden. These changes provide insight into IRIS pathogenesis and could be exploited to more effectively treat CM and prevent CM-IRIS.

*Cryptococcus neoformans,* the most common cause of adult meningitis in sub-Saharan Africa [1], causes 20%–25% of AIDS-related deaths [2, 3]. Despite this substantial burden of disease with cryptococcal meningitis (CM), the mechanisms of immune defense are not well characterized, especially at the site of infection in the human central nervous system (CNS). *Cryptococcus* is proposed to cross the blood-brain barrier through 3 mechanisms: via endothelial cells when tight junctions are damaged or weakened (paracellular) [4], via brain endothelial cells (transcytosis) [5], or within infected monocytes or macrophages (Trojan horse) [6]. Aerosol infection of mice with *Cryptococcus* spores elicits a self-limited subclinical pneumonia, accompanied sequentially by local recognition of the fungus by alveolar macrophages and neutrophils, then by monocytes and finally expansion of *Cryptococcus*-specific CD4^+^ and CD8^+^ T cells [7, 8], which parallels the immunopathogenesis in the cerebrospinal fluid (CSF) with meningitis [9]. Similarly, in healthy individuals, the initial immune response in the lung involves fungal recognition by innate immune cells and subsequent expansion of *Cryptococcus*-specific CD4^+^ T cells to control the infection [7]. In scenarios involving immunodeficiency, such as human immunodeficiency virus (HIV) infection, depletion of CD4^+^ T cells increases the likelihood of *Cryptococcus* invasion of the blood-brain barrier to cause meningoencephalitis [8, 10].

The phenotype of infiltrating immune cells at the site of infection in humans with CM is poorly characterized. Tissues from patients with CM but without HIV or other immunodeficiency show robust granulomatous inflammatory responses [11] and CSF pleocytosis [12–14], whereas among those with HIV coinfection, CSF cell counts are lower and predominantly CD8^+^ rather than CD4^+^ T cells [15, 16]. In the presence of HIV coinfection, up to 25% of patients with CM treated with antifungal and antiretroviral therapy (ART) [17] will experience paradoxical deterioration due to immune reconstitution inflammatory syndrome (IRIS) despite mycologic and virologic suppression [18]. IRIS may manifest as relapsing aseptic meningitis, increased intracranial pressure, new focal neurologic signs, intracranial cryptococcomas, lymphadenopathy, and development of abscesses [19–22]. In the majority of patients with IRIS, fungal burden decreases with antifungal therapy as evidenced by decreased cryptococcal antigen titers and sterile CSF microbiologic cultures [17, 22, 23]; however, the target tissue-specific cellular profile and activation status in CSF remain poorly characterized.

In this study, we characterized the lineage, activation, and differentiation of mononuclear cells that migrate across the blood-brain barrier in HIV-infected patients upon initial presentation with CM and at the time of CM-IRIS to better understand the localized host response in IRIS to *C. neoformans* in this immunocompromised population.

## MATERIALS AND METHODS

### Study Subjects

Study participants were prospectively enrolled in the (1) Cryptococcal Optimal Antiretroviral Timing (COAT) trial (Clinicaltrials.gov: NCT01075152), a randomized strategy trial assessing the optimal timing of ART initiation in CM [24], or in the (2) Neurological Outcomes on ART (NOAT) study, a prospective observational cohort of HIV-infected persons with clinical meningitis [25]. CSF and blood were collected from subjects screened sequentially at Mulago National Referral Hospital in Kampala, Uganda. Inclusion criteria for both cohorts included documented HIV infection, being ART naive, age ≥18 years, and clinical evidence of meningitis. Written informed consent was obtained from participants or their surrogates. Institutional review board approval was obtained from Makerere University, the University of Minnesota, and the Uganda National Council for Science and Technology.

Lumbar punctures were performed in hospitalized patients on presentation and samples evaluated with standard testing for bacteria (Gram stain and culture) and *Cryptococcus* culture and cryptococcal antigen conducted on site. Further molecular evaluation for viruses and fungi was performed on cryopreserved CSF (Supplementary Methods). Approximately 10 mL of CSF were centrifuged at 400*g* for 5 minutes to pellet cells, then cryopreserved in Roswell Park Memorial Institute medium supplemented with fetal bovine serum (20%), dimethyl sulfoxide (10%), and penicillin-streptomycin (1%) with storage in liquid nitrogen after controlled freezing.

A diagnosis of definite/probable/possible CM-IRIS was made according to the published consensus case definition [18], with external adjudication by a 3-physician panel.

### CSF Flow Cytometry

**Figure 1.**
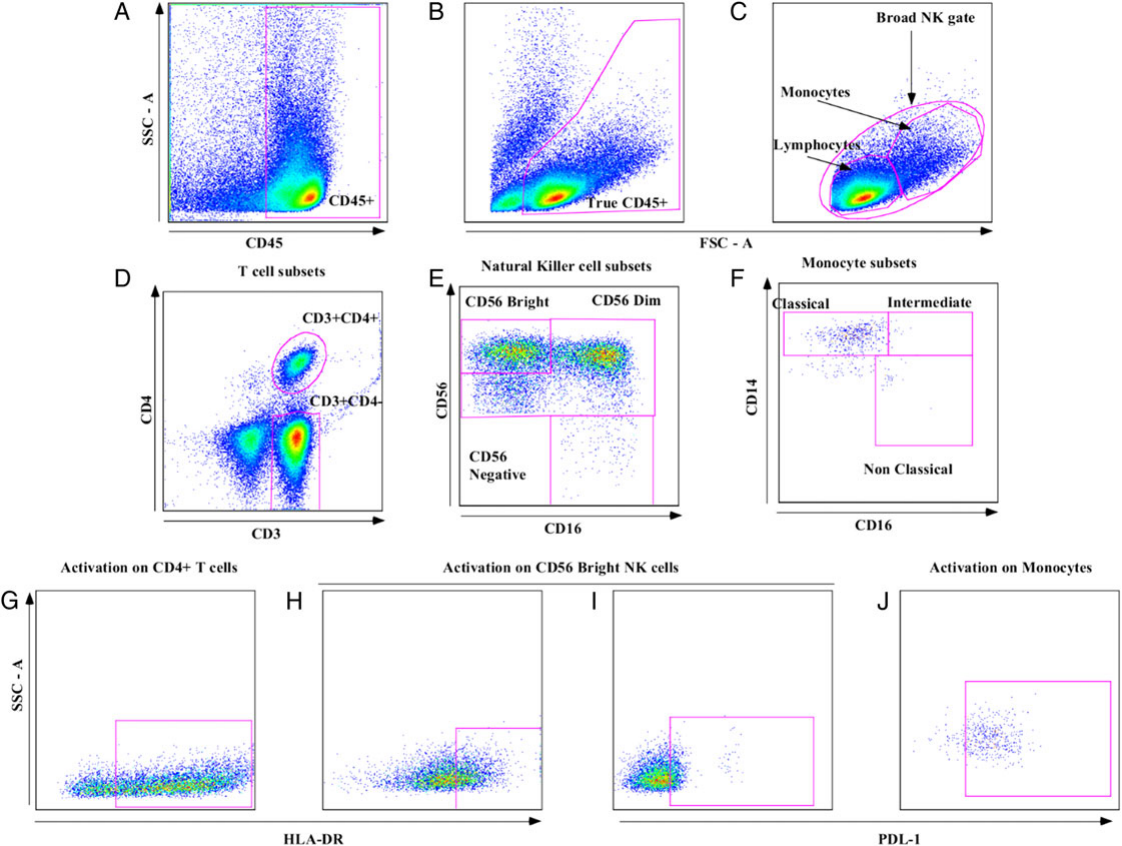
Flow cytometry gating strategy for cerebrospinal fluid (CSF) cells. Analytic gating of the flow cytometry data. *A*, White blood cells in CSF were separated from cryptococcal cells using a CD45 gate. *B*, Neutrophils and debris were separated from CD45^+^ cells on the basis of forward-scatter area (FSC-A) and side-scatter area (SSC-A). *C*, Broad natural killer (NK) cell gate, monocyte gate, and lymphocyte populations were selected. *D*, CD3^+^ T-cell subset populations were identified. *E*, NK-cell subsets were then identified based on differential expression of CD16/56. *F*, Monocyte subsets were defined based on differential CD14/16 expression. *G*, Activation by HLA-DR expression on T cells, here showing expression on CD4^+^ T cells. *H*, HLA-DR expression on NK cells, showing expression on the CD56^bright^ subset. *I*, Programmed death ligand 1 (PD-L1) expression on NK cells, showing expression on CD56^bright^ subset. *J*, PD-L1 expression on monocytes, showing expression on classic monocytes.

Polychromatic flow cytometry was performed on thawed CSF cell samples collected at screening (day 0; n = 40), day 14 (n = 21) of antifungal therapy, and at the CM-IRIS event (n = 10). Immunophenotyping of CSF white blood cells (WBCs) was performed based on panleukocyte marker CD45^+^ (Figure 1*A* and *B*), differential gating based on size and granularity (Figure 1*C*), and lineage markers for T cells (CD3^+^/CD4^+^ or CD3^+^/CD4^−^), the latter referred to as *CD8+ T cells* because >90% of this subset are CD8^+^, natural killer (NK) cell (CD3^−^4^−^CD56^+^CD16^+/−^) subsets (Figure 1*E*), monocytes (CD3^−^CD4^low^CD56^−^) and monocyte subsets based on expression of CD14 and CD16 (Figure 1*F*), as defined in Supplementary Methods. We characterized activation by expression of HLA-DR for CD3^+^ and NK cells and programmed death ligand 1 (PD-L1) for each lineage. Cells were stained with commercial monoclonal antibodies^fluorochrome^ (clone) reactive with CD3^V500^ (clone UCHT1), CD4^V450^ (clone RPA-T4; BD Horizon); CD14^FITC^ (clone M5E2, CD45^APC−Cy7^ clone 2D1, CD274/PD-L1^PE^ (clone MIH1, CD56^PerCPCy5.5^ (clone B159, CD8^APC-Cy7^ (clone SK1; BD Pharmingen); HLA-DR^PE-Cy7^ (clone LN3; eBioscience) and CD16^APC^ (clone 3G8; Biolegend) (where APC represents allophycocyanin; Cy5.5, cyanine 5.5; Cy7, cyanine 7; FITC, fluorescein isothiocyanate; PE, phycoerythrin; PerCP, peridinin chlorophyll). Fluorescence minus 1 controls were prepared on blood samples to set gates for HLA-DR and PD-L1.

### Statistical Analysis

Data were analyzed using SAS software (version 9.3; SAS Institute). Comparisons of paired groups were evaluated with the nonparametric Wilcoxon signed rank test. Cell phenotype and activation variables were compared at different time points with the nonparametric Mann–Whitney rank sum test. Statistical significance was defined at *P* < .05.

## RESULTS

### Participants

**Figure 2.**
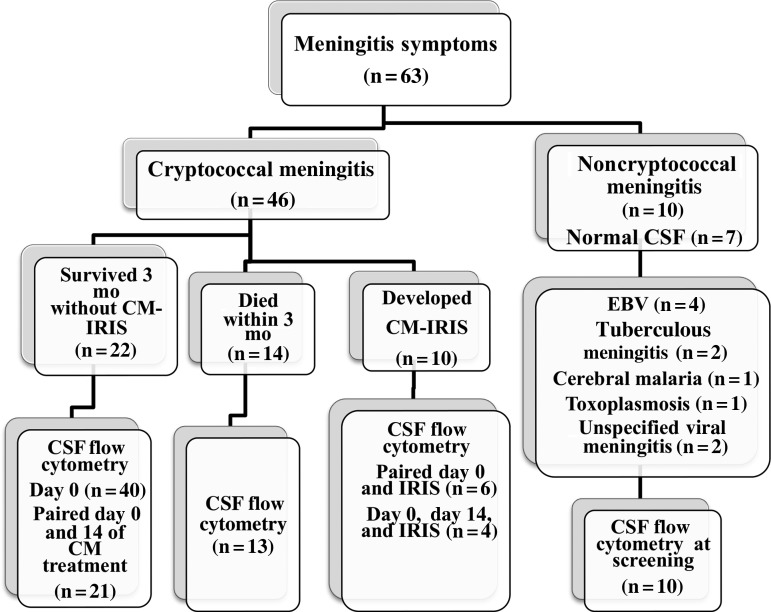
Study flow diagram. Distribution of subjects with suspected meningitis and etiology of meningitis in a nested cohort of human immunodeficiency virus-infected subjects in the Cryptococcal Optimal Antiretroviral Timing (COAT) trial and Neurological Outcomes on ART (NOAT) study in Mulago National Referral Hospital, Kampala, Uganda. Abbreviations: CM, cryptococcal meningitis; CSF, cerebrospinal fluid; EBV, Epstein-Barr virus; IRIS, with immune reconstitution inflammatory syndrome.

Sixty-three HIV-infected subjects presenting with symptoms of meningitis consented to have a lumbar puncture performed and were entered into this study. Of these, CM was diagnosed in 46 subjects, a control group of 10 subjects had noncryptococcal meningitis (NCM) and nonbacterial meningitis, and 7 subjects had normal CSF. Age, sex, and neurologic symptoms were similar in the CM and NCM groups (Table 1). All subjects with CM began ART within 6 weeks of diagnosis (zidovudine, lamivudine, and efavirenz) after induction therapy with intravenous amphotericin B (0.7–1 mg/kg/d) for 2 weeks and oral fluconazole (800 mg/d) for approximately 5 weeks, later decreased to 400 mg/d for 8 weeks and 200 mg/d thereafter. Among the 46 subjects with CM, 10 (22%) were adjudicated as having CM-IRIS during follow-up (Figure 2), 22 (48%) survived without CM-IRIS, and 14 (30%) died within 3 months of CM diagnosis. Forty subjects with CM had sufficient baseline CSF specimens available for analysis at baseline, and 21 subjects at day 14. Among subjects with CM-IRIS, 6 of 10 had CSF cells analyzed at both CM diagnosis and CM-IRIS, and 4 of these 6 subjects had CSF cells analyzed at day 14 of CM treatment. The median time from ART initiation to IRIS was 110 days (interquartile range [IQR], 73–227 days).

**Table 1.** Demographic Characteristics of HIV-Infected Subjects in Kampala, Uganda, at Enrollment With CM and NCM Meningitis and Day 14 of CM Therapy^a^

Characteristic	CM (n = 40)	NCM (n = 10)^b^
Age, y	37 (30–40)	34 (31–36)
Females, No. (%)	25 (63)	4 (40)
Symptoms, No. (%)		
Headache	38 (95)	10 (100)
Fever	31 (78)	10 (100)
Neck pain	30 (75)	9 (90)
Glasgow Coma Score <15^c^	13 (33)	8 (80)
3-mo mortality, No. (%)	14 (35)	4 (44)
Blood values at enrollment		
CD4^+^ T cells/µL	16 (6–70)	290 (51–557)^d^
CD8^+^ T cells/µL	283 (177–428)	513 (210–1239)^d^
CD4/CD8 ratio	0.08 (0.04–0.12)	0.56 (0.14–0.88)^d^
Plasma HIV RNA, log_10_ copies/mL^e^		
Day 0	5.3 (4.8–5.4)	
IRIS event	2.6 (2.0–2.6)	
CSF values at enrollment		
WBCs/µL	25 (16–83)	36 (11–678)
LFA CrAg titer^f^		
Day 0	7200 (1512–8096)	0
Day 14	1000 (512–2000)	0
Protein, mg/dL	94 (62–187)	253 (156–352)
CSF CD4/CD8 ratio	0.06 (0.03–0.12)	0.20 (0.05–0.72)
Cryptococcal CFUs/mL^g^		
Day 0	180 000 (30 400–350 000)	0
Day 14	0 (0–30)	0

Abbreviations: CFUs, colony-forming units in quantitative culture; CM, cryptococcal meningitis; CrAg, cryptococcal antigen; CSF, cerebrospinal fluid; HIV, human immunodeficiency virus; IRIS, immune reconstitution inflammatory syndrome; LFA, lateral flow assay; NCM, noncryptococcal meningitis; WBCs, white blood cells.

^a^ Data were available from 26 subjects. Unless otherwise specified, data represent medians (interquartile ranges).

^b^ Of these subjects, 4 had Epstein Barr Virus in CSF, 2 had tuberculous meningitis, 1 had cerebral malaria, 1 had toxoplasmosis, and 2 had viral meningitis.

^c^ Glasgow Coma Scores ranged from 4 to 15.

^d^ Twelve subjects did not have CD4/CD8 measured in the hospital.

^e^ Wilcoxon rank sum test was used to compare viral loads at CM diagnosis and at the IRIS event (*P* < .002).

^f^ Paired signed rank test was used to compare median CrAg titers at screening and at day 14 of CM treatment for 23 subjects (*P* < .001).

^g^ Paired signed rank test was used to compare median quantitative culture values at screening and at day 14 of CM treatment for 30 subjects (*P* < .001).

Cryptococcal antigen titers decreased significantly from diagnosis (day 0) to day 14 of CM amphotericin therapy (*P* < .001) as did the number of cryptococcal colony-forming units per milliliter during this period (*P* < .001) (Table 1). Subjects presenting with CM had severe CD4^+^ T-cell depletion in peripheral blood and viral load, decreased by approximately 3 log_10_ copies/mL from ART initiation to time of IRIS (Table 1).

### Mononuclear Cell Phenotype in CSF With CM Versus NCM

A microbiologic diagnosis was established in 10 subjects with NCM (Figure 2). The numbers of CSF leukocytes of 40 adults with CM at diagnosis did not differ from those in 10 patients with NCM (*P* = .57) (Table 1). CD8^+^ (CD3^+^CD4^−^) T cells constituted the predominant cell type in CSF among subjects with CM at day 0 (median, 72%; IQR, 57%–78%) and day 14 (median, 78%; IQR, 70%–86%) of antifungal therapy, comparable to that in subjects with NCM at presentation. At baseline, CD4^+^ T cells were higher among subjects with NCM (median, 13.6%; IQR, 1%–6%) compared with the CM group (median, 3.5%; IQR, 5%–29%; *P* = .01), whereas NK cells were present at comparable frequencies in patients with or without CM.

The CSF WBC counts (decreased during 2 weeks of antifungal therapy, from a median of 25 cells/µL (IQR, 16–83 cells/µL) at day 0 to 8 cells/µL (IQR, 3–19 cells/µL) at day 14 (*P* < .001), as did the proportion of CD4^+^ T cells. A small minority population of monocytes was present in CSF at both time points. The majority of monocytes were of the classic phenotype (or subset) (CD14^++^CD16^−^), with a median of 68% (IQR, 52%–89%) at day 0 of CM treatment, 73% (IQR, 57%–97%) at day 14, and 40% for NCM (IQR, 17%–67%). NK-cell subsets defined by CD56 and CD16 expression were also similar in each group at baseline (data not shown).

### Activation of Mononuclear Cells in CSF

**Figure 3.**
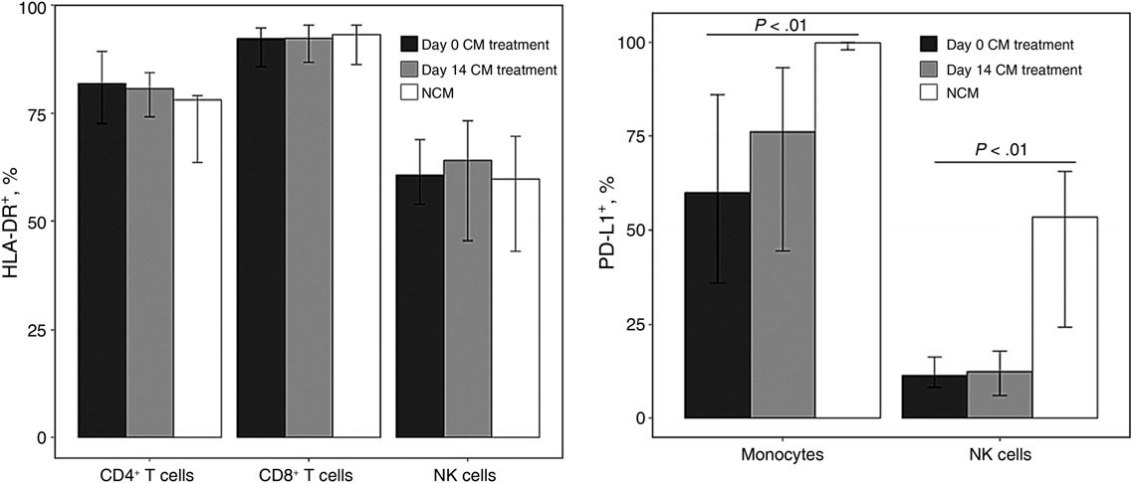
Cellular activation in cerebrospinal fluid (CSF) among subjects with cryptococcal meningitis (CM) and noncryptococcal meningitis (NCM). Activation of CD4^+^, CD8^+^, and natural killer (NK) cells in CSF is shown by HLA-DR expression and programmed death ligand 1 (PD-L1) expression on monocytes and NK cells. CSF was analyzed with flow cytometry at day 0 and day 14 of CM treatment among subjects with CM (n = 21) or NCM (n = 16) at meningitis diagnosis.

The vast majority of CSF T cells and NK cells were activated in patients with or without CM meningitis, as determined by expression of HLA-DR (Figure 3). In contrast, the frequency of expression of PD-L1 on monocytes was high in both groups at baseline, but highest in the NCM group (Figure 3), and in both classic and intermediate monocyte subsets (data not shown). A similar increase in PD-L1 was evident on NK cells in the NCM population. The high frequency of HLA-DR expression on T cells and NK cells did not change significantly during CM treatment, nor did PD-L1 expression on monocytes and NK cells (Figure 3).

### Lymphocyte Activation in CSF With CM-IRIS

Ten of 46 subjects (21%) with CM developed IRIS in the first 6 months of ART. Paired CSF samples were available from 6 of these subjects at both day 0 of CM treatment and at CM-IRIS diagnosis (Tables 2 and 3). Neither CSF WBC count nor CSF protein values differed from day 0 to the time of IRIS. Although the high proportion of CD8^+^ T cells remained stable from treatment initiation to the time of CM-IRIS, the frequency of CD4^+^ T cells increased (albeit not significantly) during this period, whereas NK cells declined (*P* = .03). These changes in lineage distribution among the subjects with paired samples were consistent with those in the larger unmatched groups comparing lineage (data not shown) and NK-cell activation at day 14 of CM treatment and CM-IRIS (Supplementary Figure 3). HLA-DR expression on CD4^+^ and CD8^+^ T cells remained elevated but stable at CM-IRIS compared with day 0 (Table 2), whereas PD-L1 expression increased significantly on NK cells overall, particularly in the CD56^dim^ NK-cell subset (terminally differentiated precursors of CD56^bright^ NK cells).

**Table 2.** CSF Parameters and Lymphocyte Phenotypes at CM Diagnosis and During CM-IRIS^a^

Parameters and Phenotypes	Day 0 of CM (n = 6)	CM-IRIS Event (n = 6)	*P* Value^b^
WBCs/µL	18 (14.0–58.0)	17 (3.5–33.5)	.31
CSF protein, mg/dL	91 (65–187)	20 (20–87)	.31
CD4^+^ T cells, %	2.2 (0.9–6.4)	22.7 (16.5–25.8)	.06
CD8^+^ T cells, %	63.6 (57.6–72.3)	66.1 (61.3–70.6)	.31
CD4/CD8 ratio	0.04 (0.01–0.08)	0.33 (0.25–0.38)	.09
HLA-DR expression, %			
CD4^+^ T cells	79.9 (70.6–94.5)	78.4 (72.5–79.5)	.31
CD8^+^ T cells	88.7 (87.7–92.7)	90.5 (89.5–91.7)	.62
NK cells, %	6.6 (4.0–7.9)	0.8 (0.3–1.2)	.03^c^
NK-cell subset			
CD56^bright^	39.0 (31.7–49.2)	30.3 (10.2–43.4)	.56
CD56^dim^	49.6 (42.1–55.1)	46.3 (41.9–80.0)	.31
CD56^−^	6.6 (2.9–17.4)	8.0 (0.0–15.2)	>.99
PD-L1 expression, %			
All NK cells	11.9 (8.0–25.3)	61.6 (44.3–100.0)	.03^c^
CD56^bright^	17.9 (11.7–30.1)	49.2 (46.9–51.4)	.16
CD56^dim^	3.7 (2.0–16.1)	54.8 (36.9–100.0)	.03^c^
CD56^−^	20.2 (16.7–34.0)	52.8 (0.0–63.6)	.44

Abbreviations: CM, cryptococcal meningitis; CSF, cerebrospinal fluid; IRIS, immune reconstitution inflammatory syndrome; NK, natural killer; PD-L1, programmed death ligand 1; WBCs, white blood cells.

^a^ All data represent medians (interquartile ranges).

^b^ Medians were compared using a signed rank test for paired observations.

^c^ Significant difference (*P* < .05).

**Table 3.** Monocyte Subsets and Activation in CSF at CM Diagnosis and CM-IRIS Events^a^

Monocyte	At CM Diagnosis (n = 6)	At CM-IRIS Event (n = 6)	*P* Value^b^
Monocytes, %	1.5 (0.7–2.0)	0.6 (0.2–1.1)	.06
Monocyte subsets, %			
Classic (CD14^++^CD16^−^)	75.0 (69.6–89.7)	13.5 (0.0–26.3)	.06
Intermediate (CD14^++^CD16^+^)	22.5 (10.0–23.9)	73.6 (47.9–92.3)	.03^c^
Nonclassic (CD14^+^CD16^++^)	2.3 (0.2–5.3)	6. 2 (3.7–23.9)	.56
PD-L1 expression, %			
All monocytes	68.8 (30.1–97.0)	96.9 (93.0–100.0)	.09
Classic	62.4 (27.9–96.6)	82.2 (33.9–95.5)	>.99
Intermediate	86.0 (48.9–97.2)	98.2 (96.0–100.0)	.06
Nonclassic	78.9 (50.0–97.3)	98.0 (94.6–100.0)	.06

Abbreviations: CM, cryptococcal meningitis; CSF, cerebrospinal fluid; IRIS, immune reconstitution inflammatory syndrome; PD-L1, programmed death ligand 1.

^a^ Data represent medians (interquartile ranges).

^b^ Median values were compared using a signed rank test for paired observations. ^c^Significant difference (*P* < .05).

Although the proportion of monocytes in CSF was stable, these monocytes showed a striking maturation from the predominant CD14^++^CD16^−^ classic subset at baseline to a more proinflammatory intermediate phenotype (CD14^++^CD16^+^) at IRIS (Table 3). Moreover, expression of PD-L1 on monocytes increased as well, particularly in the intermediate and nonclassic subsets (Supplementary Figures 1 and 2). This pattern of cell evolution with CM-IRIS was recapitulated in the larger unmatched cohort comparing lineage and activation among 17 subjects at CM diagnosis and day 14 of CM treatment and 4 subjects at CM-IRIS diagnosis. Thus, although the absolute number of leukocytes at IRIS was similar to the number at initial CM, the cellular constituents did change, as did their activation status with CM-IRIS. We considered whether these changes were unique to the site of disease in the local CSF compartment or indicative of a more generalized systemic response with ART.

### Cell Frequencies and Activation Differ Between Blood and CSF at CM-IRIS

**Figure 4.**
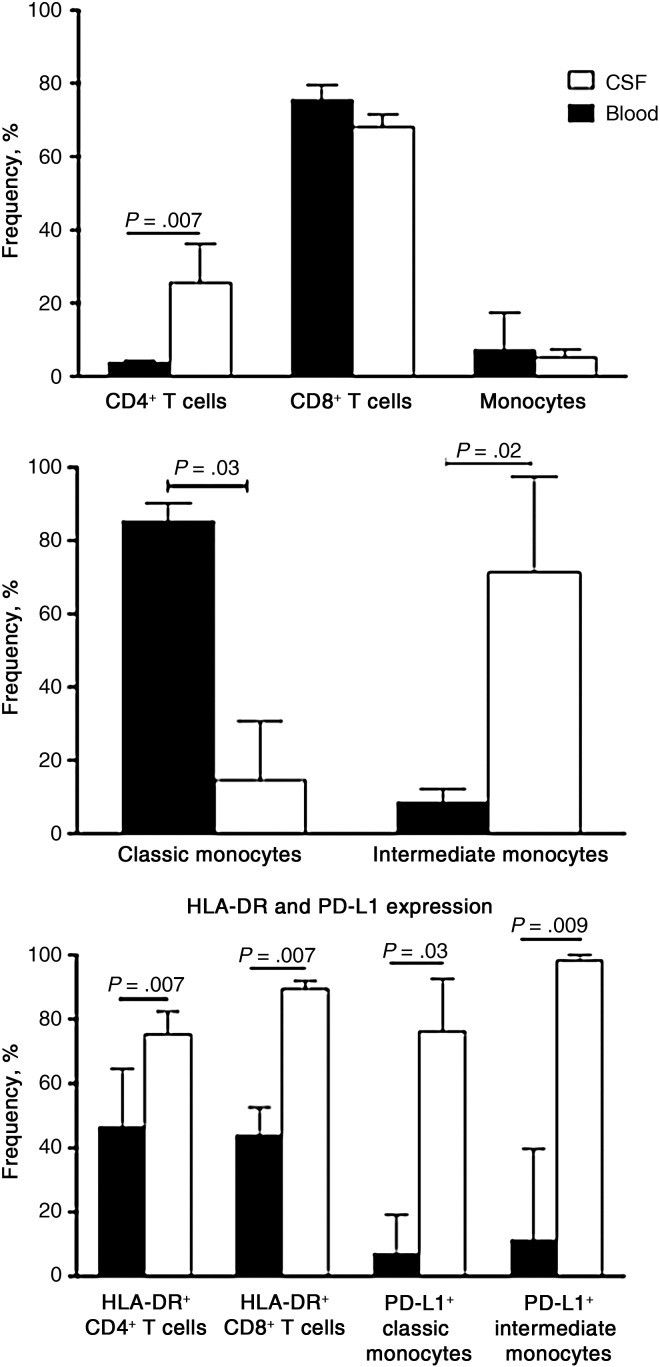
Cell phenotype and activation in matched blood and cerebrospinal fluid (CSF) at diagnosis of cryptococcal meningitis with immune reconstitution inflammatory syndrome (CM-IRIS). Cell lineages in the CSF and peripheral blood compartments (*top panel*) and monocyte subsets (*middle panel*) are shown for 6 subjects at the diagnosis of CM-IRIS. The frequency of natural killer cells (not shown) was similar. Bottom panel shows HLA-DR expression on T cells and programmed death ligand 1 (PD-L1) expression on monocyte subsets.

At the time of CM-IRIS, the frequencies and activation of lymphocytes were distinctly different in CSF and blood. Although CD8^+^ T cells were the predominant cell type in the 2 compartments, the proportion of CD4^+^ T cells was significantly higher in CSF at CM-IRIS than in blood (Figure 4). The median change in CD4^+^ T cells (from baseline to IRIS) in blood was 3.0% (IQR, 2.5%–4.5%), compared with 23% (IQR, 14%–28%) in CSF, (*P* = .06), suggesting that a greater increase in the CD4^+^ T-cell change occurred in CSF compared with blood, although this difference was not statistically significant. We also found no significant correlation between the CD4^+^ T-cell changes in blood compared with those in CSF (*r* = 0.43; *P* = .43), suggesting that these CD4^+^ T-cell changes were independent and probably compartment specific.

Consistent with these differences, the classic subset (CD14^++^CD16^−^) comprised 86% of monocytes in blood (IQR, 83%–89%) but only 13% (IQR, 0%–28%) in CSF; intermediate monocytes had higher frequencies in CSF than in blood, suggesting that IRIS represents a localized response at the site of infection rather than a more generalized response to ART (Figure 4). In line with these disproportionate cell lineages, T-cell activation (by HLA-DR expression) was significantly greater on both CD4^+^ and CD8^+^ T cells in CSF compared with blood, as was PD-L1 expression on classic and intermediate monocytes (Figure 4). The more efficient cytokine NK subset, the CD56^bright^ NK cells, tended to have higher frequencies in CSF than blood (data not shown). We demonstrate distinct phenotypic and activation changes involving both the adaptive and innate immune systems, which could play a role in the immunopathology occurring during CM-IRIS events.

## DISCUSSION

We characterized the evolution of cellular infiltrates at the site of CM infection, the CSF, from the time of diagnosis when the host is most immunosuppressed, soon after ART initiation and as immune integrity is being restored at the time of CM-IRIS in patients with advanced HIV coinfection. The number of immune cells in the CSF was quite limited in these severely lymphopenic patients despite a high fungal burden, comparable to that in patients without CM. The predominance of highly activated but apparently ineffective CD8^+^ T cells at presentation was supplemented by an increasing number of activated CD4^+^ T cells into the CSF as the fungal burden declined with antifungal therapy. The source of this enhanced immune integrity may be ART initiation as well as the evolving maturation of monocytes and the activation of both monocytes and NK cells in the CSF. However, a consequence of this proposed increased immune integrity at this local site may be the development of neurologic symptoms in the confined CNS compartment that manifest as CM-IRIS.

Both the low numbers of CSF cells and the predominance of CD8^+^ T cells in the CSF are consistent with those described elsewhere in murine models and human asymptomatic HIV infection [16, 26]. The low cell numbers could also be related to persisting cryptococcal antigen (at CM-IRIS), which is known to inhibit leukocyte migration by modulating expression of L-selectin on T cells [27, 28]. In contrast, healthy HIV-seronegative adults have very few cells in the CSF, and CD4^+^ T cells represent the majority of these few lymphocytes [16, 29]. Despite unremarkable changes in CSF profiles with antifungal treatment and ART initiation at 2 weeks, those who develop CM-IRIS show a nonsignificant increase in CSF CD4^+^ T cells and a statistically significant increase in the frequency of intermediate monocyte subsets. The associated increased expression of the immunosuppressive protein PD-L1 on both the monocyte and NK-cell subsets may reflect an exaggerated activated state. Indeed, PD-L1 is highly expressed on monocytes and macrophages during inflammatory conditions, such as septic shock and rheumatoid arthritis [30–32]. Alternatively, this ligand may serve as a counterregulatory mechanism to limit the increasingly exuberant CD4^+^ T-cell responses, which may drive monocyte maturation and activation, as well as stimulate NK-cell activity.

Missing from the development of this evolving paradigm are the ontogeny of CD4^+^ T cells, monocytes, NK cells, and their activation among patients who resolved their infections successfully with antifungal therapy and ART and those who died early in the course of this fungal infection. Characterizing the natural history of the immune response in the CSF from these latter groups over time would clarify whether CM-IRIS represents an overexuberant pathologic response to persistent antigen or the development of an increasingly normal or healthy functional response to cryptococcal antigens under the influence of ART-associated recovery of immune function.

Our results from matched CSF and peripheral blood analyses reveal that the CM-IRIS–associated changes in cellular distribution, phenotype, and surface receptor/ligand expression in the CSF compartment are distinct from and more exuberant than those in the systemic circulation. Results from studies of IRIS syndromes with other infections do not provide this unique opportunity to study the immunopathology where these processes are actually occurring in real time. Indeed, as shown here, the characteristics of immune cells in the peripheral circulation, although convenient to sample and where cryptococcal antigen also persists, represent an inaccurate or certainly an incomplete view of responses to fungal and, likely, mycobacterial (eg, tuberculosis) [33] or viral (eg, hepatitis C) [34] antigens occurring at specific tissue sites, such as the CNS, lung, or liver, all common sites of IRIS.

During CM-IRIS, the changes in CSF cell lineages and activation profiles after the initiation of ART may represent an evolving and increasingly effective CM-specific response [35]. The transition to or influx of intermediate monocytes and up-regulation of PD-L1 on CD56^dim^ NK cells were findings unique to CM-IRIS. These changes, particularly among the monocyte subsets, were not a consequence of amphotericin B, which has immunomodulatory properties, because the changes were not reliably present from day 0 to day 14 of amphotericin therapy but rather occurred well after discontinuation of amphotericin [36].

Our observed increase in intermediate monocytes at CM-IRIS is compatible with the reported relative abundance of chemokines (including CCL2 and CCL3) that promote monocyte trafficking into the CSF [15] and elevated innate cytokines including interleukin 1R, 6, and 8, tumor necrosis factor, and granulocyte–colony stimulating factor, which we have demonstrated elsewhere in blood and CSF [22, 37]. Moreover, the shift from classic (involved largely in phagocytosis and preferentially producing the immunosuppressive cytokine, interleukin 10) to proinflammatory intermediate monocytes (known to possess the highest expression of surface markers for antigen processing and presentation and produce the highest amounts of proinflammatory cytokines) probably represents a response to the inflammatory milieu present in the CNS during CM-IRIS. These changes in monocyte distribution in the CSF during CM-IRIS also support the data showing that intermediate monocytes contribute to an inflammatory systemic environment predisposing to serious non-AIDS events through secretion of proinflammatory mediators [38]. The skewed distribution of monocyte subsets suggests involvement of the innate immune system in the dysregulated response during CM-IRIS, as proposed by Barber et al [39] and recently demonstrated in tuberculosis-associated IRIS [40]. Although the classic monocyte subset may replace intermediate monocytes with successful antifungal therapy [41], we identify the opposite trend when CM-IRIS develops, highlighting the distinct local environment in the setting of this paradoxical complication of ART.

The trend to increased frequency of CD4^+^ T cells from day 14 of CM treatment until the development of CM-IRIS may reflect peripheral immune recovery in response to ART, during which naive and memory T cells may preferentially traffic into the CNS [42, 43]. It is also plausible that the influx of CD4^+^ T cells may reflect an expansion of CNS-resident effector-memory CD4^+^ T cells, consistent with the observed trend to higher interleukin 7 production with IRIS, in response to residual cryptococcal antigen as reported with other forms of IRIS, including tuberculosis-associated IRIS [44–46]. In either scenario, the milieu in CSF is clearly distinct from that in the blood in terms of cellular distributions and activation profiles. Further studies are needed to determine the antigen specificity of these CD4^+^ and CD8^+^ T cells in the CSF, which were not evaluated in our study. The small sample size was a limitation in drawing firm conclusions on associations between phenotype, activation and mortality. However, the multilineage characterization of cells in the CSF from the initiation of ART to the time of IRIS is an instructive feature of this study that requires validation in a larger cohort.

Antonelli and colleagues [46] have described high frequencies of HLA-DR^+^CD4^+^ T cells with increased expression of PD-1 on CD4^+^ and CD8^+^ T cells in blood during IRIS in the setting of diverse coinfections. Our data suggest that such activation is higher yet in the CSF compartment during CM-IRIS. A plausible hypothesis is that the interaction between PD-L1 on the innate cells with PD-1 expressed on T cells may support the initiation of a negative feedback response, to limit T-cell activation and the immunopathology triggered by exaggerated T-cell immune responses during CM-IRIS [22].

In conclusion, our findings in CSF support the hypothesis that, although immune responses appear attenuated at the time of presentation with CM among patients with very advanced HIV disease, both adaptive and innate immune axes participate in the immunopathogenesis of CM-IRIS. How the balance of adaptive immune activation and innate regulatory mechanisms are served by the apparent influx of highly activated CD4^+^ T cells and the evolution of classic to intermediate monocytes requires further investigation. We therefore hypothesize that future therapeutic intervention studies to treat or prevent CM-IRIS could exploit these distinct cellular changes as immunologic end points.

## Supplementary Data

Supplementary materials are available at *The Journal of Infectious Diseases* online (http://jid.oxfordjournals.org). Supplementary materials consist of data provided by the author that are published to benefit the reader. The posted materials are not copyedited. The contents of all supplementary data are the sole responsibility of the authors. Questions or messages regarding errors should be addressed to the author.

Supplementary Data
