# Supplementary material for: Cellular Immune Activation in Cerebrospinal Fluid From Ugandans With Cryptococcal Meningitis and Immune Reconstitution Inflammatory Syndrome
Source: J Infect Dis. 2014 Dec 9;211(10):1597–606. doi: 10.1093/infdis/jiu664 (PMC4407762; doi:10.1093/infdis/jiu664)
Supplement: Supplementary Data [file supp_211_10_1597__index.html]

Cellular Immune Activation in Cerebrospinal Fluid from Ugandans with Cryptococcal Meningitis and Immune Reconstitution Inflammatory Syndrome — Cellular Immune Activation in Cerebrospinal Fluid From Ugandans With Cryptococcal Meningitis and Immune Reconstitution Inflammatory Syndrome — Cellular Immune Activation in Cerebrospinal Fluid From Ugandans With Cryptococcal Meningitis and Immune Reconstitution Inflammatory Syndrome — Supplementary Data 

# Cellular Immune Activation in Cerebrospinal Fluid From Ugandans With Cryptococcal Meningitis and Immune Reconstitution Inflammatory Syndrome

## Supplementary Data

Supplementary Data

**Files in this Data Supplement:**

- Supplementary Data - Docx file
- Supplementary Figure 1 - tif file
- Supplementary Figure 2 - tif file
- Supplementary Figure 3 - tif file
