# Supplementary material for: Cellular Immune Activation in Cerebrospinal Fluid From Ugandans With Cryptococcal Meningitis and Immune Reconstitution Inflammatory Syndrome
Source: J Infect Dis. 2014 Dec 9;211(10):1597–606. doi: 10.1093/infdis/jiu664 (PMC4407762; doi:10.1093/infdis/jiu664)
Supplement: Supplementary Data [file supp_jiu664_jiu664supp.docx]

**Supplemental Methods**

**CSF Diagnostic Evaluation**

CSF diagnostic evaluation included: WBC and differential, protein, cryptococcal antigen (Immy, Norman, Oklahoma), India ink, quantitative fungal culture, Gram’s Stain, bacterial culture, and stain for acid fast bacilli (AFB). Xpert MTB/RIF assay (Cepheid, Sunnyvale, CA) was performed at physician discretion (n=63), using the manufacturer’s standard protocol.

Subjects negative for cryptococcosis had additional testing in the U.S. on cryopreserved (-80ºC) samples shipped on dry ice (-20ºC). 16s rRNA PCR was performed at the University of Minnesota to detect potential culture-negative bacterial meningitis. qPCR was performed for herpes simplex (HSV-1/2), cytomegalovirus (CMV), Epstein-Barr (EBV), *Toxoplasma gondii* and RT-PCR for enterovirus at the Minnesota Department of Health (MDH). Additionally, the Ibis PLEX-ID (Abbott) Broad Viral 1 Assay was performed. This involved a proprietary system able to detect Herpesviruses, Adenoviruses, Parvovirus B19, Enteroviruses and Polyomaviruses through a broad range mass spectrometry platform. Neurosyphilis testing was performed at MDH using VDRL microflocculation. Arboviral meningoencephalitis evaluation was performed in collaboration with Centers for Disease Control and Prevention by culture, genus-specific PCR amplification, and serology to evaluate for West Nile, Yellow Fever, Dengue, Chikungunya, and Zika viruses.

Subjects with confirmed cryptococcal meningitis received Amphotericin (0.7-1mg/kg/day) for 2 weeks coupled with fluconazole 800mg/day for 5 weeks. Thereafter fluconazole was reduced to 400mg/day for 8 weeks, and then secondary prophylaxis 200mg/day was given. A median of three lumbar punctures was performed for intracranial pressure control.

**Comparison of phenotype and activation - Day 14 of CM Treatment vs CM-IRIS**

We compared CSF samples from 17 subjects at day 14 of CM treatment to CSF from 7 independent subjects (without a day 14 CSF sample) at CM-IRIS. CD8^+^ T cells remained predominant at both time points with a marginal decrease at CM-IRIS. Significant changes in the frequencies of CD4^+^ T cells and NK cells at CM-IRIS were observed. Of note, CD4^+^ T cells increased at time of CM-IRIS while a decrease in NK cells was evident at CM-IRIS. A decreased frequency of classical monocytes was noted at CM-IRIS with intermediate monocytes increasing from day 14 to CM-IRIS.

A significant increase in the proportion of non-classical monocytes, CD56^dim^, CD56^neg^ and NK cells expressing PD-L1 at CM-IRIS was observed (Supplementary Figure S3)***.***

**Figure S1. Cell phenotypes in CSF at day 0, day 14 and at CM-IRIS.** Mononuclear cell lineage **(**CD4^+^, CD8^+^ T-cells, NK cells, monocytes and the proportion of Classical and Intermediate monocytes) in CSF among subjects with CSF analyzed at day 0 (n=17), day 14 (n=17) of CM treatment and at CM-IRIS event (n= 4). The black triangles represent data points for the 4 subjects with CSF samples at each time. Bars represent grouped median values.

**Figure S2. PD-L1 expression on NK cells, CD56^bri^ NK subset and monocytes in CSF.** PD-L1 expression on NK cells, CD56^bri^ NK subset and monocytes in CSF among subjects with CSF analyzed at day 0 (n=17), day 14 (n=17) of CM treatment and at CM-IRIS event (n= 4). The black triangles represent data points for the 4 subjects with CSF samples at each time (upper figure). Bars represent grouped median values.

**Figure S3. Activation of NK subsets at CM-IRIS.** Comparison of HLA-DR and PD-L1 expression on NK cell subsets among 17 subjects on day 14 of CM treatment and 7 independent subjects at CM-IRIS. Medians were compared using a Wilcoxon ranksum test for independent samples. X-axis labels for both graphs are shown on the bottom graph.
