# Supplementary figures and images for: Cellular Immune Activation in Cerebrospinal Fluid From Ugandans With Cryptococcal Meningitis and Immune Reconstitution Inflammatory Syndrome
Source: J Infect Dis. 2014 Dec 9;211(10):1597–606. doi: 10.1093/infdis/jiu664 (PMC4407762; doi:10.1093/infdis/jiu664)

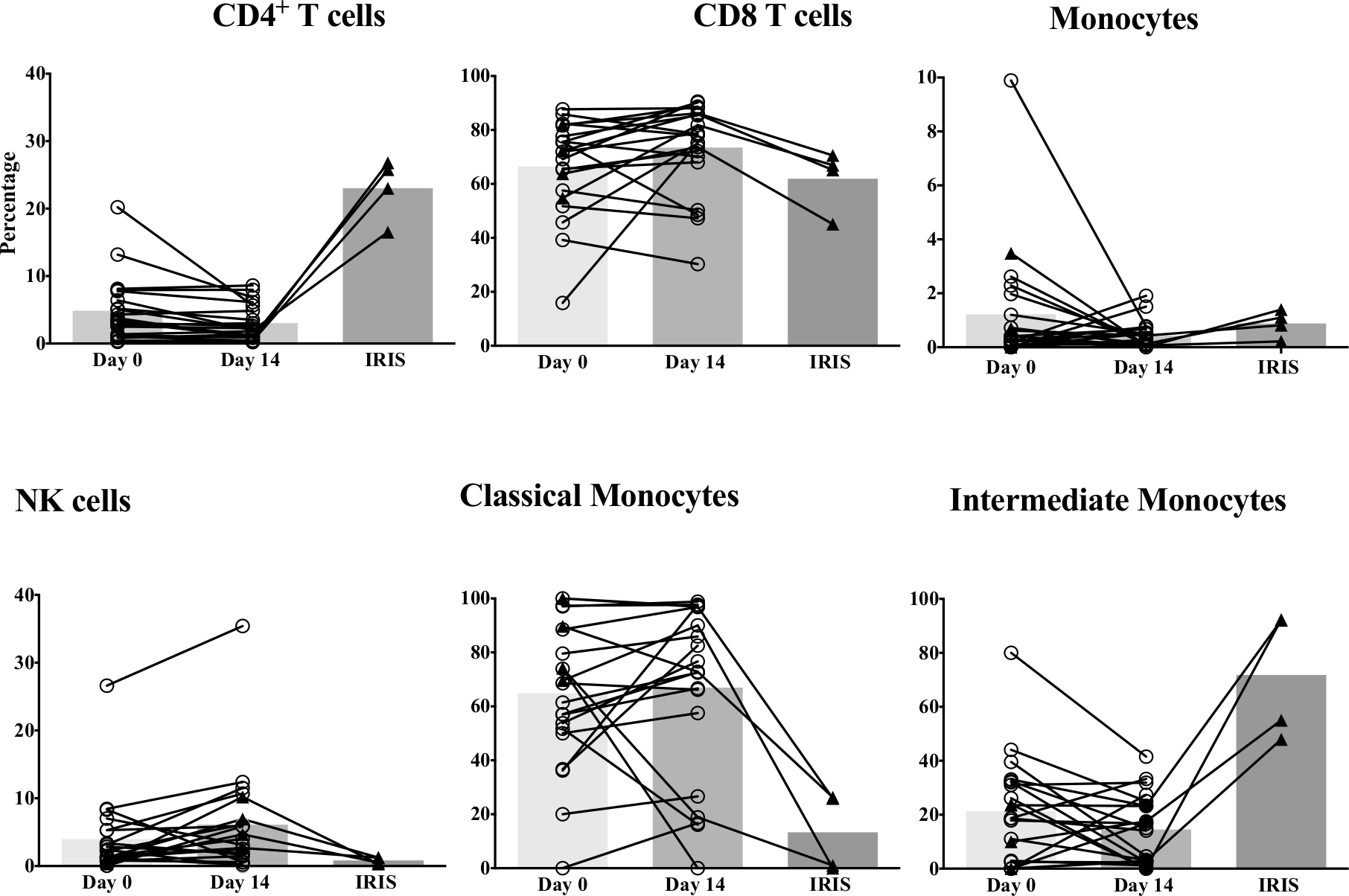

Supplement: Supplementary Data [file supp_jiu664_jiu664supp_fig1.tif]

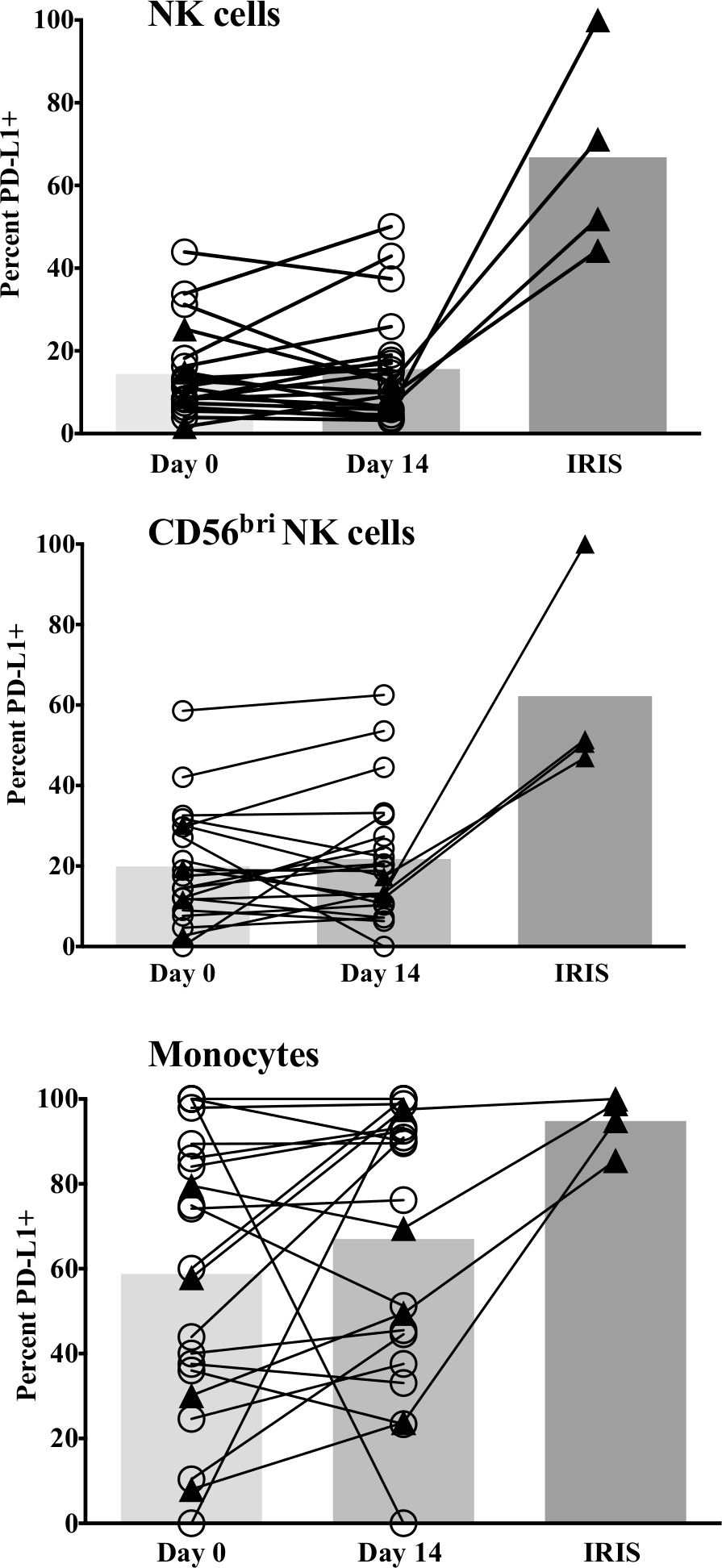

Supplement: Supplementary Data [file supp_jiu664_jiu664supp_fig2.tif]

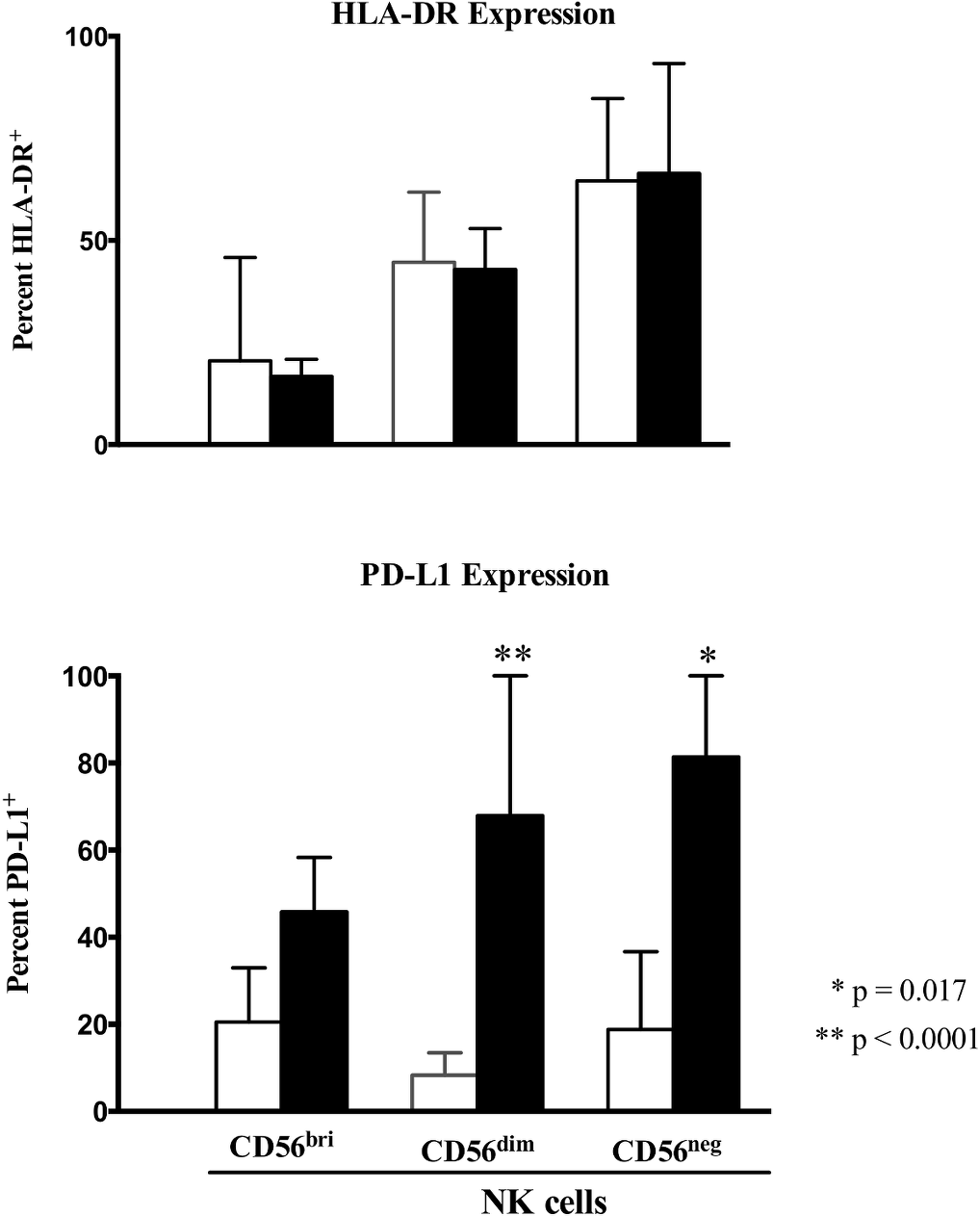

Supplement: Supplementary Data [file supp_jiu664_jiu664supp_fig3.tif]
